# Sex-related disparities in vehicle crash injury and hemodynamics

**DOI:** 10.3389/fpubh.2024.1331313

**Published:** 2024-03-15

**Authors:** Susan Cronn, Karthik Somasundaram, Klaus Driesslein, Carissa W. Tomas, Frank Pintar

**Affiliations:** ^1^Comprehensive Injury Center, Medical College of Wisconsin, Milwaukee, WI, United States; ^2^Division of Trauma and Acute Care Surgery, Department of Surgery, Medical College of Wisconsin, Milwaukee, WI, United States; ^3^Neurosurgery Department, Medical College of Wisconsin, Milwaukee, WI, United States; ^4^Joint Department of Biomedical Engineering, Marquette University/Medical College of Wisconsin, Milwaukee, WI, United States; ^5^VA Medical Center-Research, Milwaukee, WI, United States

**Keywords:** crash safety, equity in research, traumatic injury, vehicle crash, shock index, sex differences

## Abstract

**Objective:**

Multiple studies evaluate relative risk of female vs. male crash injury; clinical data may offer a more direct injury-specific evaluation of sex disparity in vehicle safety. This study sought to evaluate trauma injury patterns in a large trauma database to identify sex-related differences in crash injury victims.

**Methods:**

Data on lap and shoulder belt wearing patients age 16 and up with abdominal and pelvic injuries from 2018 to 2021 were extracted from the National Trauma Data Bank for descriptive analysis using injuries, vital signs, International Classification of Disease (ICD) coding, age, and injury severity using AIS (Abbreviated Injury Scale) and ISS (Injury Severity Score). Multiple linear regression was used to assess the relationship of shock index (SI) and ISS, sex, age, and sex^*^age interaction. Regression analysis was performed on multiple injury regions to assess patient characteristics related to increased shock index.

**Results:**

Sex, age, and ISS are strongly related to shock index for most injury regions. Women had greater overall SI than men, even in less severe injuries; women had greater numbers of pelvis and liver injuries across severity categories; men had greater numbers of injury in other abdominal/pelvis injury regions.

**Conclusions:**

Female crash injury victims' tendency for higher (AIS) severity of pelvis and liver injuries may relate to how their bodies interact with safety equipment. Females are entering shock states (SI > 1.0) with lesser injury severity (ISS) than male crash injury victims, which may suggest that female crash patients are somehow more susceptible to compromised hemodynamics than males. These findings indicate an urgent need to conduct vehicle crash injury research within a sex-equity framework; evaluating sex-related clinical data may hold the key to reducing disparities in vehicle crash injury.

## Introduction

The global burden of traffic injury has been reduced by innovations in vehicle safety design, but not all demographics have benefitted equally from this protection. Women (we will use this term to discuss our biological sex related study, with the understanding of and respect for the range of gender expression that does not correlate with biological sex) may be more vulnerable to risk of certain types of injury in vehicle crashes, yet safety features are largely based on testing with male-representative dummies. Atwood et al. (1) demonstrated greater relative fatality risk (on average, 2.9% higher fatality risk for female front row occupants vs. male) for females in vehicles with the newest generation of safety equipment, but the differences between male and female occupants' fatality risk fluctuate across age groups. Stigson et al. (2) report a greater risk of permanent medical impairment (PMI) in females compared to males, and countermeasures designed to mitigate this risk (specific to “neck” region injury, which is the region most associated with PMI) were not equally effective in men and women (3). Nutbeam et al. (4) found that female patients were more frequently entrapped after crash, and that entrapped male and female patients had differing injury patterns. As such, we cannot be certain that current vehicle safety standards in testing, equipment, and crashworthiness accurately reflect how women experience vehicle crashes; the current body of literature suggests that current methods in crash testing may not sufficiently account for male/female body differences.

Much of the literature evaluating sex differences in crash injury discusses relative risk and crash/occupants characteristics with significant effort toward comparing crashworthiness, crash severity, and adjusting for confounding factors that can affect estimated impact of sex on crash injury. In a recent IRCOBI conference, Brumbelow (5) asserts that “It is important to identify how non-physiological risk factors may affect injury risk estimates for females and males in order to encourage the most robust and effective countermeasures.” Brumbelow (5) also considers that investigating how differences in vehicles and crashes between men and women may reveal how these factors confound estimates for relative and fatality risk.

Acknowledging the difficulty in fully accounting for confounding factors is important in an accurate assessment of how men and women are injured in vehicle crashes. Clear representation of the problem of sex-related injury disparity is critical in prioritizing research, design, and allocating funding. The level of complexity of the issue, however, makes this clear representation challenging; true matched pairs comparisons are nearly impossible to achieve. We posit that by evaluating sex-related injury patterns using clinical data, we will demonstrate where and how male/female differences exist as real patient outcomes, regardless of how previous literature has estimated and quantified sex-related risk.

Atwood et al.'s (1) recent evaluation of the Fatality Analysis Reporting System (FARS) found that recent model year vehicles (2010–2020) with optimal occupant protection systems have reduced estimated female fatality risk relative to males to 5.8%; though this is an improvement, it still indicates disparity between sexes. Liu and Subramian (6) estimate the odds of a female occupant's severe injury likelihood as 1.25 times that of a male occupant. Males are more likely to engage in risky behaviors like speeding, driving while intoxicated, etc., increasing their overall likelihood of crash, death, and serious injury but even controlling for these factors, women are significantly more likely to suffer serious injuries due to vehicle crashes (7). Though some studies attribute differences in injury and fatality risk between sexes to driving patterns, behavior, and vehicle size, attempts to control for these factors in describing relative risk have not included physical stature, body mass, or other physiologic differences associated with sex (7).

A recent study by Brumbelow and Jermakian (7) discusses differences in injury severity between side and front crashes as well as differences in extremity injuries, and concludes that current vehicle safety testing has reduced injury risk to both sexes, perhaps more to female occupants. However, despite careful controlling for as many crash severity factors as possible, they posit that there are multiple sex-related properties as yet unknown, unmeasured, or unaccounted for within retrospective crash data analyses which may or may not be able to identify female vulnerability to (lower extremity) injury (7). Craig et al. (8) combined multiple crash-related databases to account for a broad range of crash types, crash variables, and occupant characteristics in an analysis of sex-based odds differences in crash outcomes. This study demonstrates the complexity of the issue, and concluded that:

“increased or decreased odds of injury for females vs. males is dependent on the type of injury and associated severity, the associated crash type, and other relevant independent variables significantly associated with the respective injury outcomes” (8).

Further, they found that in multiple models, female and male occupants were approximately equal in number of cases where each held the higher odds of injury (8). However, limitations of the study did not allow for some elements of analysis that may be relevant in comparisons of sex-related differences in crash injury outcomes—delta V, post-crash factors, or occupant characteristics like BMI, behavior, or vehicle selection (8).

To ultimately make cars that are safe for all bodies, we must isolate the risk factors which are truly due to male/female physiological differences and evaluate which elements need to be represented in crash testing. Though vehicle safety has improved overall in the past decades, this improved protection may not apply to all occupants equally. Abrams and Bass (9) posit that “there may be unobserved trends in the injury patterns, and therefore outcomes, between male and female occupants.” The objective of this study is to evaluate the disparities in abdominal and pelvic male/female injury patterns through a clinical lens; this novel approach evaluating the NTDB allows for analysis of injury patterns in trauma patients after vehicle crash. By reviewing patient injury data, this study demonstrates how injuries correlate with sex, how male and female patients are affected by these injuries, and how injury patterns demonstrate the clinical picture of known sex-related disparities in crash injury.

## Materials and methods

### Data source

The National Trauma Data Bank (NTDB) is the largest aggregation of trauma data in the USA (10). It is maintained by the American College of Surgeons (ACS) for the purposes of injury surveillance, hospital benchmarking, research and quality improvement (10). The NTDB includes extensive patient-and injury-related information from pre-hospital to discharge disposition entered by trained data registrars using established data definitions and standards. Inclusion in the NTDB is based on clinical coding for traumatic injuries. The data are audited as part of the ACS trauma center verification program, which ensures data integrity and quality (10).

### Study design

Data from the NTDB from 2018 to 2021 for patients 16 years and older were considered for analysis. This timeframe was chosen as it represents the most recently available data and also includes modern auto safety features available in newer vehicles. Only patients in vehicle crashes who were wearing lap and shoulder belts were extracted, defined using International Classification of Disease (ICD) external cause codes (*N* = 125,642) (11). The initial query included age, primary external cause codes specific to traffic-related vehicle crash (V43.5, V43.6, V44.5, V44.6, V47.5, V47.6, V53.5, V53.6, V54.5, V54.6, V57.5, V57.6), and Abbreviated Injury Scale (AIS) (12) injury diagnosis codes related to abdomen and pelvis injuries (i.e., codes beginning with 54 and 856). Injuries were grouped according to 9 generalized regions (i.e., kidney, large intestine, liver, pancreas, pelvis bony, pelvis organ, small intestine, spleen, and stomach) and assessed by sex, age group, and shock index (SI). Further variables included sex, age, initial hospital systolic blood pressure, initial hospital heart rate, injury severity score (ISS), and AIS score (both designating severity of injury). AIS-2005 standards were used as those were consistently provided across all years accessed in the NTDB.

A waiver from the Institutional Review Board at the Medical College of Wisconsin was obtained for this study.

### Statistical analysis

We report a descriptive analysis of patient characteristics. Frequencies and proportions of injury by region are described relative to total injuries and relative to total injuries by sex. Sex differences in injury by region were compared using a Chi-square test on the proportions of total injury within sex. For patients who sustained multiple injuries in the same region, the injury with the highest AIS severity was retained for analysis and the less severe injuries in that region were excluded. Multiple linear regression was used to assess the relationship of SI and ISS, sex, age, and sex^*^age interaction. Coefficient estimates are reported along with 95% confidence intervals for each term in each model. ISS was used in lieu of AIS scores to account for overall injury severity (rather than just severity within the respective body region examined). ISS is intended to be an objective anatomical scoring system that quantifies injury severity by summing the squares of the AIS scores for the 3 most severely injured body regions. ISS scores range 0–75 with scores 0–9 indicating mild severity, 9–15 indicating moderate, 16–24 severe, and over 25 indicating profound injury (12). ISS is greater than 15 is usually considered major trauma (13).

Where data were available, shock index (SI) calculated by the ratio of heart rate (HR) over systolic blood pressure (SBP) was derived (*N* = 122,557; 97.5% of total sample) (14). Nine separate regressions were completed (one per injury region) to compare how patient characteristics may differ related to shock index depending on the injury sustained and a Bonferroni correction was applied to adjust for multiple comparisons. All analyses were completed in R (version 4.3.0).

## Results

### Demographic characteristics of crash injury patients

From 2018 to 2021, 56,839 patients sustained at least one abdominal or pelvis injury from a traffic-related vehicle crash and also had complete demographic and injury related information available in the database (45.2% of total vehicle crash patients). Of these patients, 28,292 (49.7%) were men and 28,547 (50.2%) were women. Patients ranged in age from 16 to 25 years (24.7%), 26 to 34 years (18.1%), 35 to 44 years (14.1%), 45 to 54 years (11.9%), 55 to 64 years (12.5%), 65 to 74 years (9.96%), 75 to 84 years (6.9%), and above 85 years (1.8%). Within age categories, male and female representation was as follows: male 51.4% and female 48.5% of age 16–25 years; male 53.5% and female 46.4% of age 26–34 years; male 52.6% and female 47.3% of age 35–44 years; male 50.2% and female 49.7% of age 45–54 years; male 47.0% and female 52.9% of age 55–64 years; male 42.9% and female 57.0% of age 65–74 years; male 40.6% and female 59.3% of age 75–84 years; and male 43.7% and female 56.2% of age 85 and greater.

### Injury patterns

Of the 56,839 patients, there were 81,459 total abdominal and pelvis injuries (Table 1). Patients sustained on average 1.43 injuries (abdomen and pelvis only, range = 1–8 injuries per patient, median = 1). Of the total number of injuries sustained, 36.9% were pelvis (bony), 16.3% spleen, 13.8% liver, 11.3% small intestine, 7.6% kidney, 5.58% pancreas, 5.35% large intestine, 3% pelvis (organ), and < 1% stomach.

**Table 1:** Patient characteristics of total abdominal and pelvis injuries (*N* = 81,459 injuries).

	***N* (%)**	**Female**	**Male**	**16–25**	**26–34**	**35–44**	**45–54**	**55–64**	**65–74**	**75–84**	**85+**
Kidney	6,191 (7.6)	2,913 (7.14)	3,278 (8.06)	1,732 (8.31)	1,015 (6.76)	862 (7.51)	739 (7.63)	791 (7.89)	589 (7.59)	375 (7.14)	88 (6.29)
Large Intestine	4,358 (5.35)	1,942 (4.76)	2,416 (5.94)	1,399 (6.71)	926 (6.17)	572 (4.99)	491 (5.07)	394 (3.93)	349 (4.50)	181 (3.45)	46 (3.29)
Liver	11,201 (13.8)	6,120 (15.0)	5,081 (12.5)	3,354 (16.08)	2,299 (15.31)	1,608 (14.02)	1,177 (12.16)	1,217 (12.14)	829 (10.68)	572 (10.90)	145 (10.36)
Pancreas	4,544 (5.58)	2,223 (5.45)	2,321 (5.71)	977 (4.69)	692 (4.61)	616 (5.37)	579 (5.98)	636 (6.34)	517 (6.66)	406 (7.73)	121 (8.64)
Pelvis (bony)	30,032 (36.9)	15,546 (38.1)	14,486 (35.6)	6,908 (33.13)	5,466 (36.41)	4,240 (36.96)	3,640 (37.60)	3,728 (37.19)	3,085 (39.73)	2,312 (44.04)	653 (46.64)
Pelvis (organ)	2,444 (3)	1,182 (2.9)	1,262 (3.1)	694 (3.33)	587 (3.91)	382 (3.33)	243 (2.51)	213 (2.12)	176 (2.27)	120 (2.29)	29 (2.07)
Small Intestine	9,192 (11.3)	4,299 (10.5)	4,893 (12.0)	2,182 (10.46)	1,611 (10.73)	1,312 (11.44)	1,204 (12.44)	1,298 (12.95)	961 (12.38)	502 (9.56)	122 (8.71)
Spleen	13,250 (16.3)	6,425 (15.8)	6,825 (16.8)	3,548 (17.01)	2,382 (15.87)	1,857 (16.19)	1,572 (16.24)	1,706 (17.02)	1,233 (15.88)	764 (14.55)	188 (13.43)
Stomach	247 (< 1)	128 (0.31)	119 (0.29)	59 (0.28)	36 (0.24)	23 (0.20)	37 (0.38)	41 (0.41)	25 (0.32)	18 (0.34)	8 (0.57)
Total	81,459 (100)	40,778 (50.1)	40,681 (49.9)	20,853 (25.6)	15,014 (18.4)	11,472 (14.1)	9,682 (11.9)	10,024 (12.3)	7,764 (9.5)	5,250 (6.4)	1,400 (1.7)

**Figure 1:**
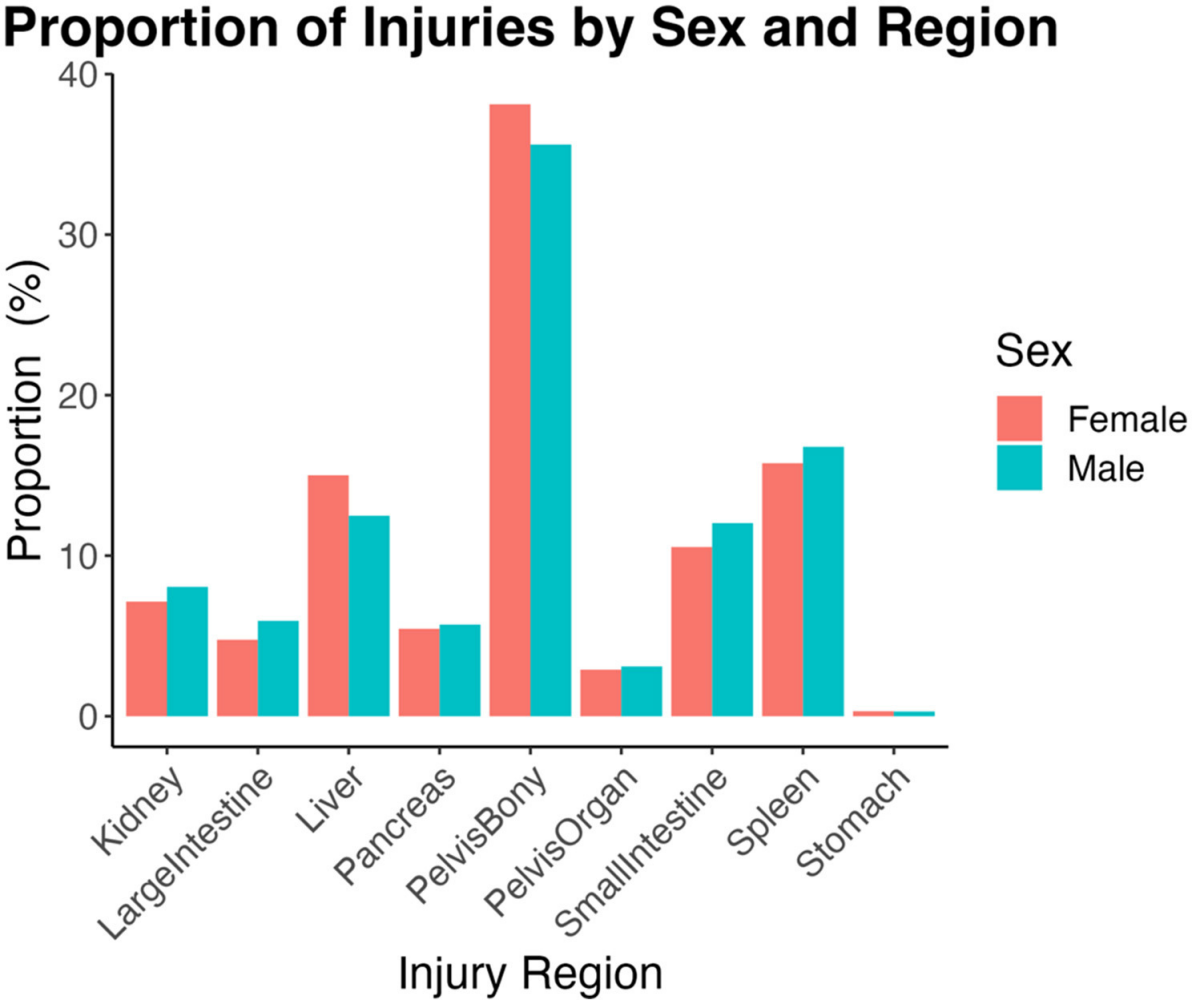
Proportions calculated relative to total injuries within each sex and then by injury region. Females sustain numerically more pelvis (bony), liver, and stomach injuries compared to men. There were no significant sex differences in rates of injury by region [$\chi_{\left(64\right)}^{2}$ = 72.0, *p* = 0.23].

For female patients, pelvis (bony) injuries were most frequent (38.1%), followed by spleen (15.8%), liver (15.0%), small intestine (10.5%), kidney (7.14%), pancreas (5.45%), large intestine (4.76%), pelvis (organ; 2.9%), and stomach (< 1%). For male patients, injury frequencies were ranked the same as female patients (Figure 1), with the most frequent being pelvis (bony; 35.6%) followed by spleen (16.8%), liver (12.5%), small intestine (12.0%), kidney (8.0%), large intestine (5.94%), pancreas (5.71%), pelvis (organ; 3.1%), and stomach (< 1%). There were no significant sex differences in proportions of injury by region [$\chi {\;}_{\left(64\right)}^{2}$ = 72.0, *p* = 0.23].

**Figure 2:**
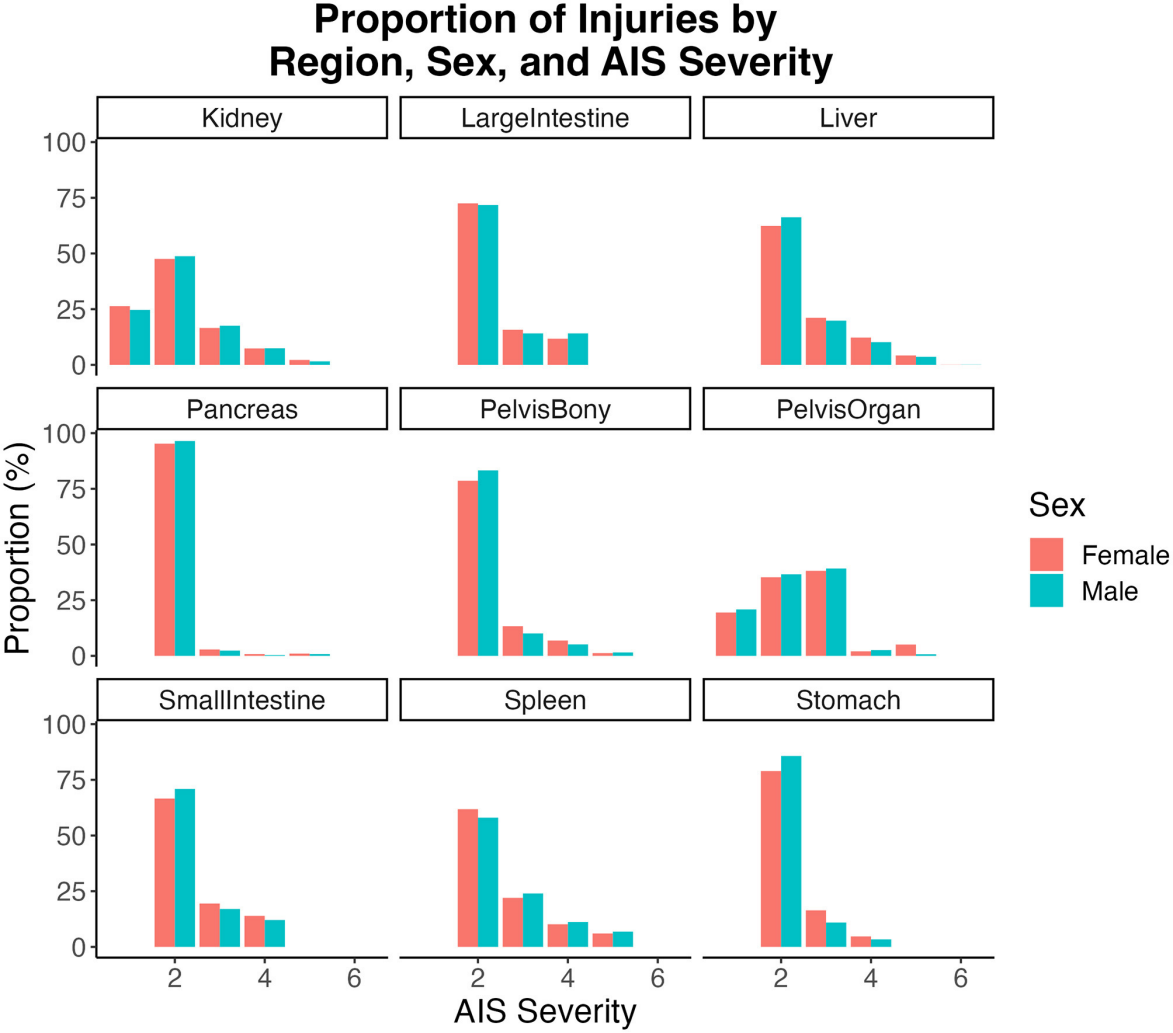
Proportions calculated within injury regions for each sex by AIS severity.

For all injury regions in both male and female patients, except pelvis (organ), AIS 2 injuries were most common (frequency/number) (Figure 2). For pelvis (organ) injuries, AIS 3 injury was most common. For injury regions where females sustained greater numbers of injuries than males [i.e., liver, pelvis (bony), and stomach], they did so across AIS severity levels 3 and 4. A similar pattern (across higher AIS severity levels) held for injury regions where males sustained more injuries [i.e., kidney, large intestine, pancreas, pelvis (organ), small intestine, and spleen] than females.

**Figure 3:**
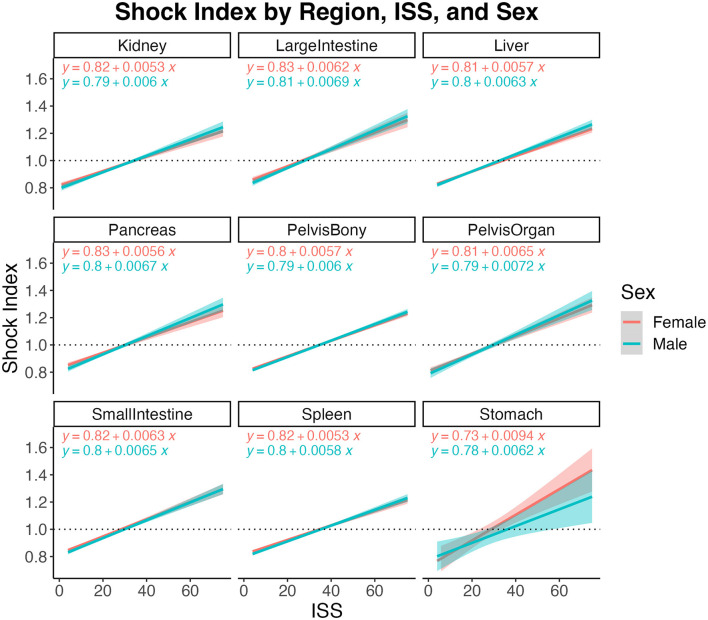
Shock index relative to injury severity scores (ISS) by sex for each injury region. Dashed horizontal line at 1.0 indicates critical clinical status where heart rate value has exceeded systolic blood pressure value. Shaded bands depict 95% confidence intervals with respective linear regression equation shown in each panel for each group.

Results of the multiple linear regression models examining SI for each injury region are reported in Table 2. For all injury regions, higher shock index was associated with greater injury severity and younger age (Table 2). When one sex had greater numbers of injuries in a region, it was consistent across all levels of severity. Females across all injury regions, except stomach, had significantly higher shock indices than males at lower ISS scores; as ISS increased, sex differences largely dissipated (Figure 3). Except for kidney, pelvis (organ), spleen, and stomach, there was a significant sex^*^age interaction for all other injury regions. Together these results suggest sex, age, and ISS are strongly related to shock index for most injury regions. Of note, in the full sample, age and ISS were significantly but very weakly correlated (*r*^2^ = 0.0002, *p* < 0.0001); in females this pattern holds (*r*^2^ = 0.0009, *p* < 0.0001), but in males there was no significant relationship (*r*^2^ = 0.000001, *p* = 0.38). This suggests age differences in ISS do not diminish sex differences. Further, we conducted a sensitivity analysis whereby we repeated the reported regressions for only those with ISS > 15, which indicates severe injury (*n* = 45,839; 49.5% female). The only differences were that sex was no longer significant for pelvis (organ) injuries, and that the sex^*^age interaction term was no longer significant for liver or large intestine injuries. Therefore, results did not substantially change as there were still significant age and sex differences across most injury regions despite examining only severe injury.

**Table 2:** Relationships of injury type with shock index via multiple linear regression.

**Injury region**	**Regression coefficients**	**Model variables**			
		**ISS**	**Sex (male)**	**Age**	**Sex (male)** ^*^ **age**
Kidney	Estimate	**0.009** ^*^	**−0.08** ^*^	**−0.003** ^*^	0.001
	CI	(0.0084, 0.0095)	(−0.114, −0.05)	(−0.0039, −0.0028)	(0.0003, 0.0017)
Large intestine	Estimate	**0.01** ^*^	**−0.11** ^*^	**−0.002** ^*^	**0.002** ^*^
	CI	(0.010, 0.012)	(−0.156, −0.079)	(−0.0035, −0.0021)	(0.0011, 0.0032)
Liver	Estimate	**0.009** ^*^	**−0.075** ^*^	**−0.002** ^*^	**0.001** ^*^
	CI	(0.009, 0.010)	(−0.097, −0.053)	(−0.0031, −0.0024)	(0.0007, 0.0018)
Pancreas	Estimate	**0.01** ^*^	**−0.065** ^*^	**−0.002** ^*^	**0.0005** ^*^
	CI	(0.009, 0.01)	(−0.10, −0.026)	(−0.0033, −0.0020)	(−0.00036, 0.0014)
Pelvis (bony)	Estimate	**0.0092** ^*^	**−0.11** ^*^	**−0.003** ^*^	**0.001** ^*^
	CI	(0.0090, 0.0095)	(−0.12, −0.10)	(−0.0034, −0.0030)	(0.0015, 0.0021)
Pelvis (organ)	Estimate	**0.01** ^*^	**−0.10** ^*^	**−0.003** ^*^	0.001
	CI	(0.010, 0.012)	(−0.156, −0.046)	(−0.0049, −0.0027)	(−0.0001, 0.0028)
Small Intestine	Estimate	**0.01** ^*^	**−0.10** ^*^	**−0.002** ^*^	**0.001** ^*^
	CI	(0.010, 0.011)	(−0.13, −0.07)	(−0.0032, −0.0023)	(0.0009, 0.002)
Spleen	Estimate	**0.008** ^*^	**−0.07** ^*^	**−0.002** ^*^	0.0008
	CI	(0.0082, 0.0091)	(−0.10, −0.05)	(−0.003, −0.002)	(0.0002, 0.001)
Stomach	Estimate	**0.01** ^*^	−0.10	−0.003	0.001
	CI	(0.0093, 0.015)	(−0.29, 0.08)	(−0.006, −0.0009)	(−0.003, 0.005)

Regressions run separately for each injury region. Estimate, unstandardized regression coefficient; CI, 95% confidence interval for each estimate; ISS, injury severity score; ^*^p < 0.05 after Bonferroni adjustment. Bold values are unstandardized regression coefficient. Statistically significant positive values indicate the variable's increase or positive association with increase in SI; statistically significant negative values indicate the variables decrease or negative association with increase in SI.

## Discussion

In the 56,839 patients meeting study criteria, women and men were represented fairly evenly in overall proportion of crashes. For all patients, age group 16–25 was 23.2% of the total and the next highest group was 26–34 at 16.9%. These two groups combined were 40.1%, with age groups 35–44, 45–54, 55–64, and 65–74 represented nearly equally at 13.6, 12.4, 13.7, and 11.7%, respectively. There was a sharp drop off in crash numbers from ages 75 and above, with those groups comprising only 9.1% of the total number of crash patients. Within age groups, men were more highly represented from ages 16–44, but this equalized from ages 45–54 and then reversed, with women as the greater number of crash victims above age 55 (increasing proportions with each jump in age group). Injuries (by number or total counts) were similarly evenly distributed across male/female patients, with some exceptions: women had greater number of liver injuries in all AIS categories 2–5, greater number of bony pelvis injuries in all AIS categories 2–5, greater number of pelvis organ injuries in AIS category 5, and greater number of stomach injuries in AIS categories 3 and 4.

A critical piece of information uncovered by this group is the presence of elevated shock index in female crash victims at rates greater than in male crash victims. Women crossed the shock threshold (SI > 1.0) at a lower ISS score in all injury regions. Women also crossed the shock threshold at fewer number of total injuries than men. However, the difference in SI converged as number of total injuries increased. This difference was most pronounced in patients under the age of 30 and in patients with pelvis injury.

Shock index is a useful tool in the rapid assessment of a trauma patient (14). A quick look at HR and SBP and simple calculation (e.g., is the ratio HR:SBP > 1?) can orient the clinician to the hemodynamic status of the patient, even in an austere environment with minimal equipment. Since hemorrhagic shock is a leading cause of death during initial trauma intervention, early recognition of shock is key to timely treatment (14). Because patients can appear “normal” despite significant hemorrhagic loss due to physiologic compensation, an SI > 1.0 can provide an early alert for those patients likely to need mass transfusion, ICU admission, or other interventions to prevent morbidity and mortality (14).

The normal ratio of heart rate to systolic blood pressure ranges between 0.5 and 0.7, with some sources accepting up to < 0.9 as within the range of normal. SI is a better predictor of shock than HR and SBP separately, and since not all blood loss is visible it is critical to identify hemorrhagic shock quickly (15). With hypovolemia (less than normal amount of blood and fluid in the body's circulatory system) caused by blood loss due to injury, the initial physiological response in trauma patients is increased heart rate, which compensates for the reduction in stroke volume (how much blood the heart pumps out to the body with each heartbeat). Heart rate (beats per minute) multiplied by stroke volume (mL) equals cardiac output, which is expressed in liters per minute. This cardiac output is what supplies the body's tissues with oxygen, and perfusion of these tissues with oxygen is the normal state of a healthy patient (16).

As a traumatically injured patient's cardiac output declines due to blood loss, the body's tissues receive less oxygen than needed. This mismatch leads to multiple other compensatory mechanisms (peripheral vasoconstriction, anaerobic respiration, diversion of blood from non-critical organs to heart and brain) (16). A patient in early or class I shock (blood loss up to 15% of circulating blood volume) may demonstrate very few clinical signs that they are in trouble; elevation in HR is usually the first clue (17). As the HR rises, the SI will increase; this will continue and become more pronounced as systolic blood pressure begins to drop (which first happens consistently at 30% or greater blood volume loss) (17). It is dangerous to wait until a patient falls into a precise category of shock before taking action (hemorrhage control, transfusion, operative intervention); hemorrhagic shock is a clinical emergency which requires immediate treatment as soon as it is detected (17).

Higher SI was associated with worse injuries and with youth—the ability to compensate physiologically by increasing heart rate is stronger in younger patients. In addition, many patients above the age of 65 use medications inhibiting their ability to increase HR regardless of need (i.e., beta blockers), a variable not available in this database. Of note, even with lower injury severity scores, females had significantly higher shock indices regardless of injury region, except for stomach. This indicates either an inaccuracy in widely held standards in vital signs due to inattention to sex differences or a greater physiologic response to trauma in females, or another factor as yet unknown.

If female crash injury victims are entering shock states with lesser injury severity than male crash injury victims, this may have serious clinical implications. It suggests that female patients are somehow more susceptible to compromised hemodynamics and elevated SI after injury, which indicates a higher likelihood of transfusion, ICU stay, and mortality (14). In trauma care, clinicians consider the mechanism of injury and injury severity as context for expected patient hemodynamic status; if sex is not considered in this calculus, clinicians could be delaying identification of shock in female patients. No current trauma assessment uses sex as a data element or directive, except in injuries of pregnant people (which is focused on pregnant physiology and complications, not sex specifically). It is possible that clinical practice may need to adapt to sex-related hemodynamic differences, which will become clearer with further research.

The implications of evidence showing greater physiologic distress in women with equivalent or lesser injuries from vehicle crash than men are potentially meaningful across a number of domains. From a clinician standpoint, increasing use of shock index as a tool to assess impending worsening of clinical status may result in earlier identification of those individuals in need of intervention or may signal a need to EMS personnel for greater haste, higher level of care, or preparation of a trauma center for their arrival (16). Evaluating crash injury with the knowledge that a female patient may have higher likelihood of shock could allow trauma clinicians to risk stratify and recognize and treat shock more aggressively, potentially resulting in fewer complications. As we develop our understanding of the relationship between sex and shock index, it is possible that clinicians caring for patients in the ICU or inpatient unit may need to consider the impact of sex on hemodynamics in their treatment plans.

From a vehicle safety and design standpoint, the implication of either lesser physiologic reserve or lower resilience in female occupants, greater vulnerability to injury, and increased likelihood of increasing shock index in certain injuries/injury patterns may require a re-evaluation of current practice in how safety and design are conceived and developed. Aside from the clear imperative to move forward in average-sized female dummy evolution and use, current standards in vehicle safety must include review of the impact of current equipment on those injuries for which females have increased risk of shock. Transparent, equity-focused research and design will require a commitment to eliminating disparities in crash injury from both manufacturing and legislation, areas which have, until recently, allowed these disparities to remain unaddressed.

### Prior research and knowledge

Though research has begun to evaluate the differences in crash injury between male and female occupants, the majority of the work arises from the engineering field. This cross-disciplinary group sought to combine engineering and clinical expertise for a new perspective on how to approach reducing sex-related disparities in crash injury. By including a clinical standpoint, novel elements are integrated into existing research strategies, driving both disciplines to expand upon and amplify their understanding of the problem.

Prior research discusses the statistics related to differences in male/female crash injuries, but does not explain why they occur, nor does it examine the impact or significance of these differences, nor does it attempt to define the clinical relevance of sex-related crash injury disparities. By combining clinical and engineering-related data, contextualizing specific abdominal and pelvic injury patterns (chosen due to potential injury relationship with seat belts) could help narrow this gap in understanding. The unexpected finding of sex-related disparity in shock index provides incredible weight to the need for a convening of expertise directed toward the problem of sex-related crash injury differences, with the goal of parsing which elements of crash dynamics, human physiology, and current vehicle safety equipment are interacting to create these disparities. Further implications of investigating the clinical perspective of vehicle crash injury include discovery of how other differences in body types may contribute to unequal protection by vehicle safety equipment. There is a dearth of literature describing how height, weight, weight for height (BMI), age, and disability can be represented in current crash testing practices.

To understand possible root causes of disparity in crash injury, it is essential to briefly review the structure of legislation surrounding vehicle safety. Vehicle crash testing only requires two variations of adult-representative dummies, an average sized (50th percentile) male, and a small female (5th percentile) (18). Furthermore, the female dummy is a scaled down version of the male dummy, which means it does not account for differences in body composition, mass distribution, or muscle/bone mass, density, and strength (19). There is no requirement that vehicles are tested using an average sized female dummy, nor is there a requirement for sex differentiation in dummy design and construction, nor are there sex-differentiated injury criteria for use in testing.

### Limitations

The initial frequency analysis used to begin the process of sorting through a large dataset does not reflect the full picture of individual injury pattern. This study focused on abdominal and pelvis trauma, but there are multiple other injuries that will need consideration in the context of shock index. Trauma center participation in the NTDB is voluntary and therefore these data do not constitute registry information from all trauma centers in the U.S. Patients who died prior to emergency room presentation as well as those who did not seek or receive care from a trauma center would also not be captured in this database. Data quality for study variables are limited by the NTDB data standard. In the NTDB use of beta blockers (or other medications and conditions affecting heart rate and blood pressure) is not documented which almost certainly affected the analysis of shock indices (the likely skew of beta blockers, for instance, would be in reducing the mean of SI in higher age groups). We were also not able to use the modified shock index, due to unavailability of diastolic blood pressure to calculate Mean Arterial Pressure. We were unable to account for any contextual information regarding the vehicle crash that may affect injury patterns such as direction, speed, or force of the crash, and the position of the patient in the vehicle relative to impact.

## Conclusion/interpretation of findings

- Women and men appear to have some differences in crash-related abdomen and pelvis injury, both in actual injury pattern and in their physiologic response to the trauma.
- Though many of these differences are attenuated in different age groups, the finding that women have greater risk of shock across multiple injury types, severities, and ages indicates that even in comparable situations, women may be more vulnerable to the injuries they experience.
- Injuries to bony pelvis, pelvic organs, liver, and stomach were more frequent in women than men, which may indicate a starting point for safety equipment evaluation.

With greater numbers of pelvis (bony, AIS 2–5), pelvis (organ, AIS 5), liver (AIS 2–5), and stomach injuries (AIS 3–4) in women, it is possible that some element of anatomical difference between male and female bodies is interacting with safety equipment in a way that increases these injuries. Since current vehicle safety equipment is designed for a standard male figure, the question of whether a female occupant may somehow be under-protected, either due to equipment fit (i.e., seat belt positioning) or because female occupants make out-of-standard adjustments to accommodate their size, proportions, or weight distribution (i.e., distance to steering wheel) needs further investigation. Female drivers are often considered “out-of-position,” but this designation of women as non-standard is the primary root of multiple inequities in research and design of daily-use, safety-related, or health-affecting equipment. The absence of female bodies as their own standard clearly has consequences, which in the case of vehicle crashes, can be serious and life-altering.

By elucidating the differences between male/female injury patterns and connecting them to male-preferential safety equipment, we may clear a path for research to pursue any number of vehicle occupant variations in injury and vehicle design; whether this will require improved dummy technology, computer modeling, or a combination of the two remains to be seen. Findings from the clinical approach described in this study can be used to address sex-based discrepancies in a critical area of injury-related public health, and can be used to prioritize future directions for sex-related crash injury research.

## Data availability statement

The original contributions presented in the study are included in the article/supplementary material, further inquiries can be directed to the corresponding author.

## Ethics statement

The studies involving humans were approved by IRB Medical College of Wisconsin. The studies were conducted in accordance with the local legislation and institutional requirements. Written informed consent for participation was not required from the participants or the participants' legal guardians/next of kin in accordance with the national legislation and institutional requirements.

## Author contributions

SC: Conceptualization, Data curation, Formal analysis, Funding acquisition, Investigation, Methodology, Project administration, Writing—original draft, Resources, Supervision, Writing—review & editing. KS: Formal analysis, Validation, Writing—review & editing. KD: Data curation, Formal analysis, Methodology, Writing—review & editing. CT: Data curation, Formal analysis, Methodology, Validation, Writing—original draft, Writing—review & editing. FP: Conceptualization, Formal analysis, Methodology, Supervision, Validation, Writing—review & editing.
